# Supplementary material for: Modelling the Effects of Beverage Substitution during Adolescence on Later Obesity Outcomes in Early Adulthood: Results from the Raine Study
Source: Nutrients. 2019 Dec 3;11(12):2928. doi: 10.3390/nu11122928 (PMC6950484; doi:10.3390/nu11122928)
Supplement: Supplementary file 1 [file nutrients-11-02928-s001.pdf]

Online supporting material

Table S1. Comparison of baseline characteristics between non-participants and participants from ages 14 to 22 years.

	Non-participants (n=497)		Participants (n=667)		
	Mean	SD	Mean	SD	P-value
Age (years)	14.1	0.2	14.1	0.2	0.9
Body mass index (kg/m^2)	21.5	4.2	21.1	3.9	0.027
Waist circumference (cm)	76.5	11.1	74.8	10.3	0.003
Plain water (g)	679.6	516.7	744.9	541.8	<0.0001
Tea/coffee (g)	49.6	102.3	44.0	96.6	1.0
Diet drink (g)	26.2	89.0	18.2	61.7	<0.0001
SSB (g)	348.1	340.7	271.9	282.6	<0.0001
100% fruit juice (g)	74.1	107.0	90.8	122.7	0.8
Milk(g)	392.8	273.2	394.1	317.3	0.9
Total energy intake(MJ)	9.9	3.1	9.4	2.9	<0.0001
Aerobic activity(watts)	110.2	28.6	111.7	30.1	0.2
Healthy dietary pattern z-score	-0.1	0.9	0.1	0.9	<0.0001
Western dietary pattern z-score	0.1	0.9	-0.1	0.8	<0.0001
Categorical variables	%		%		
Gender					
Males	54.2		49.5		0.115
Females	45.8		50.5		
Dietary misreporting					
Underreporters	22.5		27		0.392
Plausible reporters	65.4		62.3		
Overreporters	11.2		10.7		
Maternal education					
High	56.0		69.8		<0.0001
Low	44.0		30.2		
Family income (rank)					
0	32.3		24.6		<0.004
1	31.3		29.6		
2	20.5		22.7		
3	15.9		22.1		

Table S2. Beverage intake at age 14 years

	Consumer(n)	% Consuming	Mean	SD
Sugar-sweetened beverages (g)	627	94.0	271.6	282.5
Plain water(g)	602	90.3	744.8	541.4
Tea/coffee(g)	298	44.7	43.9	96.5
Diet drinks(g)	152	22.8	18.2	61.7
100% fruit juice (g)	477	71.5	90.9	122.6
Milk(g)	650	97.5	393.9	317.0

SD: standard deviation
